# Factors associated with hookah smoking among women: A systematic review

**DOI:** 10.18332/tpc/110586

**Published:** 2019-08-01

**Authors:** Sakineh Dadipoor, Gerjo Kok, Teamur Aghamolaei, Ali Heyrani, Mohtasham Ghaffari, Amin Ghanbarnezhad

**Affiliations:** 1 Health Education and Health Promotion, Mother and Child Welfare Research Center, Hormozgan University of Medical Sciences, Bandar Abbas, Iran; 2 Department of Work and Social Psychology, School of Psychology and Neuroscience, Maastricht University, Maastricht, Netherlands; 3 Social Determinants in Health Promotion Research Center, Hormozgan Health Institute, Hormozgan University of Medical Sciences, Bandar Abbas, Iran; 4 Environmental and Occupational Hazards Control Research Centre, School of Public Health, Shahid Beheshti University of Medical Sciences, Tehran, Iran

**Keywords:** women, hookah, waterpipe, shisha, narghile, galyan, hubble bubble

## Abstract

**INTRODUCTION:**

The present study aimed to determine factors associated with hookah smoking among women on a global scale based on a systematic review of related literature. Intervention Mapping was the guiding framework for this review.

**METHODS:**

Searches were performed in Web of Science, PubMed, Iranian databases, Elsevier, Embase, Scopus, Medline, Google Scholar, and the World Health Organization (WHO) website, using keywords related to hookah and associated terms. Studies in English or Persian, published between 1990 and 2018, were included in this review if they were available in full text and had a target population of women. Determinants of hookah smoking at the intrapersonal, interpersonal, institutional/organizational, community, and political levels, were extracted.

**RESULTS:**

Positive attitude, social-psychological needs, low perceived risk, social-cultural acceptance of hookah, easy access and lack of laws were among the reasons given for consuming hookah. Because hookah smoking is a multifactorial issue, the qualitative method alone was not sufficient to identify the determinants of hookah smoking among women. The opinions of experts in the field of smoking control had been largely neglected in the obtained studies, and most quantitative studies lacked a theoretical framework.

**CONCLUSIONS:**

To reduce the rate of hookah consumption, actions to be taken include changing women’s positive attitude toward hookah, learning to resist friends’ pressure to smoke, highlighting the unpleasantness of hookah smoking by segregating places with transparent walls within public places, showing in the virtual world that hookah smoking is socially unacceptable, limiting access to hookah tobacco products, and effectively implementing rules that restrict hookah smoking in public places.

## INTRODUCTION

The hookah (also known as waterpipe) is a device that comprises several parts through which tobacco smoke is often mixed with a fruity aroma. The main form of tobacco consumption in many countries is cigarette consumption. Yet, hookah smoking constitutes a significant portion of tobacco consumption at the global scale^1^. According to the latest World Health Organization (WHO) report, tobacco kills more than 8 million people each year. More than 7 million of those deaths are attributed to direct tobacco use while about 1.2 million are among non-smokers exposed to secondhand smoke^2^. Hookah smoking rates are reported to be 8.7% among Jordanian women^3^, 4% among Lebanese women^4^, and 4% among Pakistani women^5^. Iran is among the nations whose citizens prevalently smoke hookah. The hookah consumption rate in Iran is close to the global rate due to social acceptance, access to different tobacco flavors, and low cost of hookah materials^6^. Some studies in Iran have reported the prevalence of hookah smoking among men and women to be 1.7% to 10.9% and 0% to 16.8%, respectively. One study reported the prevalence of hookah smoking among women in the province of Sistan to be 16.8%, in Bushehr to be 14.8%, and in Hormozgan to be 10.3%^7^. The different psychological, social, biological and physiological features of women compared to men, especially concerning the tendency women show toward different drugs, and the different reaction that society shows to women’s hookah consumption, explain the more serious state of consumption among women and why it is essential to deal with this matter^8^. Hookah smoking among women is accompanied by a higher risk of preterm menstrual pause, reduced bone mineral density, infertility, and ectopic pregnancy; it is also associated with a higher rate of infant mortality and can lead to intrauterine growth restriction and the rise of certain chromosomal anomalies^9-11^. According to the findings of various studies, women who smoke before pregnancy are more likely to continue smoking during pregnancy^12^. Alberg et al.^13^ reported a higher rate of affliction with lung cancer among women than men caused by inhaling the cancerous substances within smoke^13^. In another investigation, Pascale et al.^14^ showed that the side effects of hookah smoking are greater among women than men^14^. Other investigations have further reported that women have a more positive attitude towards hookah and are more dependent on it than men^14-16^. Global statistics point to the increasing rate of hookah smoking among women compared to men^17-21^.

The rate of hookah smoking and, most importantly, the popularity of hookah smoking among women are increasing; besides, women play a salient educational and constructive role within families. Therefore, it is essential to recognize individual and environmental determinants of hookah smoking^22^ in order to prevent and reduce tobacco consumption among women and maintain the health of society and future generations. So, the present review aimed to determine what underlying factors are associated with hookah smoking among women. It is hoped that the findings of the present study will help the development of a successful plan to confront the issue.

## METHODS

According to the objective of the study, key words related to hookah smoking were selected. Then, search terms (Table 1) were constructed by using conjunctions to connect the keywords.

**Table 1:** Search terms used in the study

	Search terms*
1	‘Factors’ OR ‘Affecting’ OR ‘Risk Factor’ OR ‘Risk Factors’ OR ‘Related Factor’ OR ‘Related Factors’ OR ‘Associated Factor’ OR ‘Associated Factors’
2	‘Waterpipe smoking’ OR ‘Waterpipes’ OR ‘Smoking’ OR ‘Pipe’ OR ‘Smoking Water’ OR ‘Pipe Smoking’ OR ‘Water Smoking’ OR ‘Waterpipe Smoking’ OR ‘Smoking Waterpipe’ OR ‘Smoking pipes’ OR ‘Hookah Smoking’ OR ‘Smoking Hookah’ OR ‘Hookahs’ OR ‘Sheesha’ OR ‘Shisha’ OR ‘Shishas’ OR ‘Narghiles’ OR ‘Narghile’ OR ‘Hubble-bubble’ OR ‘Hubble’ OR ‘Narkeela’ OR ‘Bubble’ OR ‘Hubby-bubby’ OR ‘Gaza’ OR ‘Argil’ OR ‘Oriental’ OR ‘Boory’ OR ‘Glass base’ OR ‘Ghalyan’
3	‘Female’ OR ‘Woman’ OR ‘Women’ OR ‘Adolescent’ OR ‘Adolescence’ OR ‘Youth’

* The [tiab] field code was used after each free-text term to restrict query to search in the title and abstract of each article.

To find relevant literature, Web of Science, PubMed, Iranian databases, Elsevier, Embase, Scopus, Medline, Google Scholar and the WHO website were searched. A reference search of the grey literature in journals, research abstracts and conference proceedings were also conducted. Searches took place in November and December 2017 and were updated in September 2018.

### Inclusion and exclusion criteria

Articles regarding studies with a target population other than women, that reported the results of both sexes, or that focused on other types of smoking (such as cigarettes) as well as those that aimed to explore other factors (e.g. the correlation between hookah smoking and health outcomes) were excluded. Articles that failed to distinguish between hookah smoking and smoking other substances (studies that explored the effect of several substances simultaneously) and interventional studies were also excluded as well as studies that explored the correlation between demographic information and hookah smoking. All articles were in Persian or English and had been published between 1990 and 2018.

### Screening

**Figure 1:**
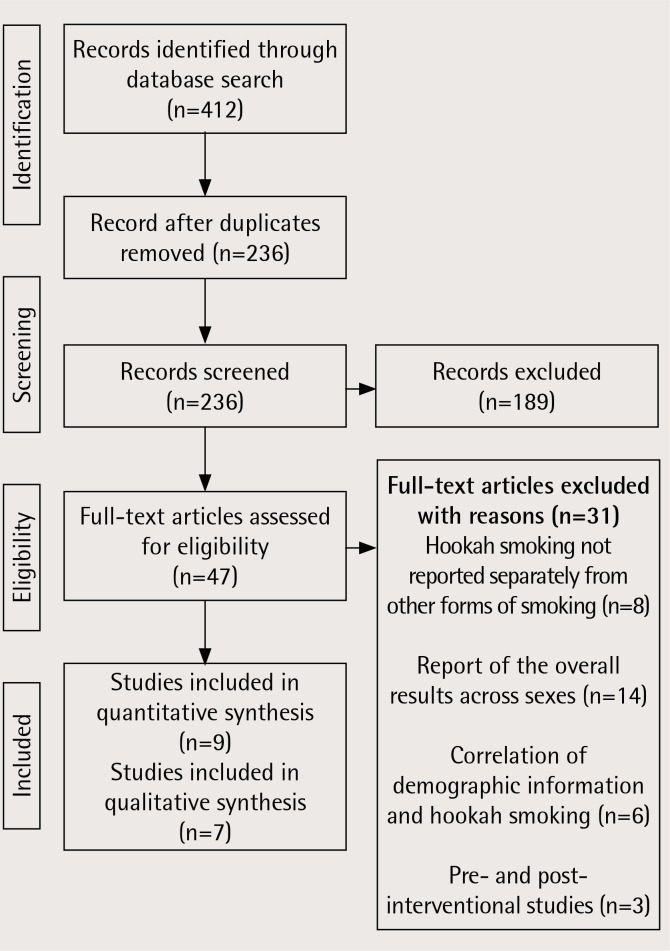
Flow chart describing literature extraction process

Two researchers performed the primary search separately, which resulted in 412 articles. Once duplicates were removed, 236 articles remained. After reading the abstracts and titles of the remaining articles, 189 records were excluded. Then the full-text of the remaining 47 articles were analyzed for eligibility. An additional 31 studies were excluded for the following reasons: hookah smoking not reported separately from other forms of smoking (n=8), report of overall results across sexes (n=14), correlation of demographic information and hookah smoking (n=6), and pre- and post-interventional studies (n=3). In total, 16 studies remained for the final analysis (Figure 1).

### Quality assessment

A quality assessment of the quantitative studies was done using the 22-item checklist ‘Strengthening the Reporting of Observational Studies in Epidemiology’ (STROBE)^21^. The articles were classified as good (scores within the range of 17–22), fair (8–16) and poor (1–7) (Supplementary file 1). Then, a critical appraisal of the qualitative studies was conducted using the checklist for qualitative research with 10 items^22^ (Supplementary file 2).

### Data extraction

Relevant data were extracted, described and entered into an Excel file. The data included: first author, year of publication, purpose of study, setting, sample size, and determinants of hookah smoking among women at the intrapersonal, interpersonal, institutional/organizational, community, and political levels.

## RESULTS

In total, 16 studies were identified, including 7 qualitative, 8 quantitative and one commentary study. The vast majority of the studies had been conducted in Asia and the US and most explored the factors involved in hookah smoking at the intrapersonal or interpersonal level. Other features and a summary of factors associated with hookah smoking are presented in Table 2.

**Table 2:** Description of included studies

*Code*	*Study YearYear Country*	*Type of study*	*Type of analysis/data collection method*	*Aim of study*	*Sample size/age*	*Determinants*	*Scores on the quality assessment*
1	Baheiraei et al.^24^ 2015 Iran	Qualitative	Content analysis in-depth individual interviews	Factors that contribute to the first hookah smoking trial by women: A qualitative study from Iran	36/24 years	**Intrapersonal:** Positive attitude, common misconception, the mutual role of psychosocial needs and gaps, popular and enlivening fashion and sensory characteristic of hookah. **Interpersonal:** Social and family facilitators.**Community:** Cultural acceptance of hookah, affordability and availability of hookah, social changes.	B
2	Sohrabzade & Parnian^8^ 2015 Iran	Qualitative	Grounded theory Semi-structured interview technique was interactive	Qualitative studies smoking hookah among girls and young women	37/not reported	**Intrapersonal:** Pleasure, fashion and prestige leisure, emotional and psychological needs. **Interpersonal:** Peer pressure, family negligence. **Community**: Extensive access. **Policy**: Absence of any rules and regulations against hookah smoking.	B
3	Afifi et al.^25^ 2013 Eastern Mediterranean	Qualitative	Thematic analysis Focus group discussions (FGDs) and key informant interviews	Explore the social norms and attitudes that lead to being a more acceptable form of tobacco smoking in women than cigarettes	38 (interview) 81 (focus group)/18–25, 26–35, 36–65 years	**Intrapersonal**: Sensory characteristics, consumerism, dependence. **Community**: Socio-cultural norms.	A
4	Baheiraei et al.^57^ 2015 Iran	Qualitative	Content analysis In-depth individual interviews	Find the role of psycho-social needs and gaps as a possible risk factor for hookah smoking initiation in women	36/15–51 years	**Intrapersonal**: Curiosity, desire for non-feminine, forbidden, negative, need for amusement and recreation.For others: to show off, attract attention, satisfy, and join others activities.	A
5	Nakkash et al.^19^ 2011 Lebanon: Beirut and Bekaa	Qualitative	Thematically analyzed Focus groups and in-depth interviews	Understand waterpipe smokers and non-smokers’ views of the reasons behind the spread of hookah	220/18–25, 26–35, 36–65 years	**Intrapersonal**: Innovations in design, and sensory qualities of hookah smoking. **Institutional /Organizational:** Media influence. **Community**: Availability, affordability. **Policy**: Control policies.	A
6	Hammal et al.^28^ 2015 Canada	Qualitative	Focus group Open-ended question and probes	Explore the cultural significance of waterpipe smoking	Not reported/18–30 years	**Intrapersonal**: Positive attitude. **Interpersonal**: Peer influence. **Community**: availability, facile access to cafes, culture.	B
7	Baheiraei et al.^30^ 2015 Iran	Qualitative	Content analysis In-depth individual interviews	Role of family members’ smoking behaviors as a possible risk factor for initiation of hookah smoking in women	36/15–51 years	**Interpersonal**: The role of family and relatives, the role of husband and his family. **Community**: Entry of hookah into homes.	A
8	Labib et al.^26^ 2007 Egypt	Quantitative	Analysis – pooled data logistic regression models	Investigated behavioral and sociodemographic factors associated with tobacco use among female university students patronizing hookah cafes	196 medical students /21 years	**Interpersonal**: Cigarettes encouraged by other females (56.6%).	12
9	Yunis et al.^23^ 2007 Beirut, Lebanon survey	Quantitative	Interviews with surveyed mothers Two stepwise logistic regression models	Describe patterns of cigarette and narghile (hubble-bubble or waterpipe) smoking before and during pregnancy and identify predictors of successful smoking cessation	3967/not reported	**Interpersonal**: Partner’s smoking habits (p<0.001).	17
10	Dar-Odeh et al.^31^ 2013 Amman, Jordan	Quantitative	Conveniently selected Chi-squared test	Narghile smoking among Jordanian educated working women: Attitudes and beliefs	96/mean age 34.2 years with age range of 25–45.5 (SD=7.8)	**Interpersonal**: Friends and relatives narghile smoking (73.7%).	11
11	Firoozabadi et al.^29^ 2015 Iran	Quantitative	Convenience and snowball sampling Anova, Pearson correlation coefficient, independent t-tests and linear regression	Examine the predicting factors affecting continued intension to waterpipe smoking (WPS) among women consumers	430/15–83 years	**Intrapersonal**: Attitude (r=0.57, p=0.000), instrumental attitude (r=0.44, p=0.000), perceived behavioral control (r=-0.17, p=0.000) was associated with continued intention of WPS. **Interpersonal**: Subjective norms (r=0.26, p=0.000).	16
12	Chaaya et al.^4^ 2004 Lebanon	Quantitative	Stratified sample Chi-squared test, Mann–Whitney test	Assess pregnant Arab women’s knowledge of chemical contents and related harmful effects of narghile and cigarettes	864/27 years	**Intrapersonal**: Knew little about the harmful ingredients of narghile, many misconceptions regarding how narghile worked or how it can produce harm. Positive attitude.	16
13	Maziak et al.^58^ 2004 Syria	Quantitative	Interviewer-administered, anonymous questionnaires Quasi-perception score	Gender and smoking status-based analysis of views regarding wastepipe and cigarette smoking	416/24 years	**Intrapersonal**: Positive perceptions, more positive attitudes.	16
14	Azab et al.^3^ 2013 North and Middle of Jordan	Quantitative	Random selection Multivariate analysis	Exposure of pregnant women to waterpipe and cigarette smoke	500/20–40 years	**Intrapersonal**: Positive attitude. **Interpersonal**: Husband smoking, friends.	17
15	Salameh et al.^14^ 2012 Lebanon	Quantitative	Multistage cluster sample Backward stepwise likelihood ratio multiple logistic regression	Evaluate whether nicotine dependence was higher in Lebanese women smokers compared with men smokers	2201/≥40 years	**Intrapersonal**: Pleasure, conviviality, and social interaction, termination of dysphoric, positive reinforcement.	18
16	Dar-Odeh & AbuHammad^27^ 2011 Arab	Quantitative commentary	Not reported	The changing trends in tobacco smoking for young Arab women: Narghile, an old habit with a liberal attitude	Not Reported	**Interpersonal**: Family, peer pressure. **Policy**: Absence of any rules and regulations against hookah smoking.	N/A

### Factors associated with hookah smoking

#### Intrapersonal factors

##### Positive attitude

Having a positive attitude was reported in both qualitative and quantitative studies as the most prevalent cause of hookah smoking among women. Other common reasons for hookah smoking included normalization of hookah use, traditional, popular and enlivening fashion of hookah consumption^8,23,24^. Also, the fruity taste and smell of flavored tobacco, visualization of the smoke, decorative feature of the waterpipe, the soothing sound of the bubbling water, and the thought that hookah smoking helps concentration, were mentioned as the contributing factors for hookah smoking^25^.

##### Psychosocial needs

Psychosocial needs also play a determining role in hookah smoking. The results of the studies indicated that curiosity, the need for amusement and recreation, a desire for non-feminine, forbidden and negative activities, a desire to show off, attracting the attention of others^8,24^, pleasure^8,14^, happiness, avoiding sadness^14^, and entertainment, were some of the factors that affect hookah smoking^25^.

##### Risk perception

A review of the related literature revealed that women perceived hookah to be healthier than cigarettes^4,24,26^.

##### Perceived lack of addiction to hookah

The literature review indicated that women believed hookah to be less addictive than cigarettes^3,24^.

### Interpersonal influences

Social norm was another reason for hookah consumption, which included social and family facilitators (friends and peers), family and relative pressure^3,8,24,27-30^, the role of a husband and his family^3,30^, and partner’s smoking habits^23,31^.

### Organizational/institutional influences

The Media are among the factors associated with hookah smoking; men and women smokers and nonsmokers perceived media, especially movies and TV series broadcast during Ramadan, to be influential in creating a positive image of hookah and its attraction^19^.

Several investigations showed that affordability and access to hookah are among factors associated with the popularity of hookah smoking among women and teenage girls^19,24,28^.

### Community influences

Several investigations reviewed in this study pointed to the social acceptance and cultural norms associated with hookah smoking^21,25,28^. In the present review, social revolutions, such as women’s entrance to the job market and belated marriage, were among the reasons given for smoking hookah^24^.

### Policy influences

In the present review, only one study investigated the political aspect of hookah smoking. In that study, the participants in the focused group discussion (FGD) maintained that certain policies should be made against tobacco consumption, especially hookah smoking. The policies suggested by the participants included forbidding hookah consumption in closed spaces such as restaurants, increased prices, restrictions on direct and indirect advertisements by mass media, and the display of warnings on hookahs like those on cigarettes^25^.

## DISCUSSION

As global statistics point to a higher rise in hookah consumption among women than men^16-20,32^, research to identify various factors associated with hookah consumption among women is essential in order to help design effective and systematic interventions. Hookah consumption is a multifactorial phenomenon with different political, social and economic factors involved. Thus, in the present review, hookah-smoking determinants within a certain framework with five levels (personal, interpersonal, organizational, social, political) were extracted.

### At the intrapersonal level

Having a positive attitude toward hookah was among the main reasons for hookah smoking in women more than in men^15,16^. A higher score of positive attitude toward hookah smoking was accompanied by a lower rate of intention to quit^33^. In their investigation, Jeihooni et al.^34^ reported that effective hookah-smoking cessation interventions should focus on the positive attitude toward hookah consumption. So, to create a negative attitude toward hookah consumption, some suggestions are: persuasive communications through videos and effective discussions, the use of messages designed to enrich positive beliefs and introduce new ones, messages within a positive/negative framework, the use of popular and positive models to highlight the disadvantages of hookah smoking, the use of cues for action including warnings in hookah smoking establishments, putting up posters in places marked by heavy traffic, showing a loathing of hookah smoking behavior in mass media, and showing how smoking hookah can negatively influence the body.

### Psychosocial needs

Also playing a determining role in hookah smoking are psychological needs. A review article referred to leisure as the main reason for smoking hookah^35^. Other investigations found that teenagers think hookah has positive effects in reducing stress, anger and depression, creating oblivion, and improving their concentration^33,34,36^.

Based on the results, another reason for smoking hookah was wrong perceptions of its harmlessness. Roskin and Aveyard^37^ reported low risk and the filtering of toxins through water as the most frequently mentioned causes of a tendency toward hookah smoking^37^. The majority of investigations showed that teenagers perceive hookah to be less harmful than cigarettes^38-42^. Based on the study findings, women also believed hookah to be less addictive than cigarettes. Other investigations showed that teenagers did not perceive hookah to be addictive, and thought they could quit hookah smoking whenever they wished^33-35,39,43,44^. Hence, such mistaken thoughts and beliefs should be taken into account and correct knowledge should be increased through mass media and education.

### At the interpersonal level

Social norm was another reason for hookah consumption. Some research among female university students reported peer pressure to be the strongest predictor of smoking^45^. Similarly, inability to resist peer temptation and difficulty avoiding smoking hookah at friendly gatherings were the main reasons for having a tendency toward hookah smoking^33^. A body of research indicated that family and friends can play a key role in encouraging hookah consumption^40,46^. To raise the level of personal control, the following recommendations should be considered: behavior enrichment, physical and mental improvement, behavior self-monitoring, model development through a step-by-step recorded video presentation on how to resist peer pressure, family education through mass media on their pivotal role in inclination toward hookahs, raising awareness about the disadvantages of hookah, and enhancing effective communication between parents and children.

### At the organizational level

According to the study results, easy access to hookah and its low cost were among other factors involved in hookah consumption at an organizational level. Participants, in a study by Roskin et al.^37^, maintained that the lower cost of hookah compared to other entertaining substances had made them more interested in hookah smoking^37^. Other investigations also referred to easy access to hookah as among the reasons why people prefer it^21,35,47^. Another investigation referred to the increasing number of hookah smoking establishments that served hookahs as a stimulus for publicizing hookah consumption^48^. Moreover, it was shown that the number of hookah establishments surrounding universities result in high prevalence of hookah smoking among them^49^. Thus, health planners and policy makers should consider the growing number of restaurants and hookah-smoking establishments that provide hookah services.

### At the community level

Based on the study findings, a lack of embarrassment about smoking hookah has led to the increased prevalence of this behavior. A body of research revealed that a high percentage of hookah smokers perceive hookah smoking as socially acceptable and believe that other people perceive it positively also^39,46,48^. Another factor associated with hookah smoking among women is that they regard it as a cultural norm. Some hookah smokers, especially adults, believe that hookah smoking is rooted in public culture and traditions^21^. Some researchers have referred to changes in culture and traditions as reasons why women are more interested in hookah smoking^50^. Therefore, society must act in a way to convince individuals that hookah smoking is an inappropriate behavior similar to cigarette smoking. Other possible strategies to decrease the popularity of hookah smoking in society are: highlighting the unpleasantness of hookah smoking by segregating places with transparent walls within public places, showing hookah smoking to be socially unacceptable through social networks, and showing TV series in which negative characters smoke hookahs. Another possibility is that there might be different people across cultures with different religious beliefs. Religion can probably influence hookah consumption behavior. Some research has demonstrated the role that religion plays in reducing the rate of hookah consumption^51,52^; however, this issue needs further research.

### At the political level

Although many countries follow strict tobacco-control policies and regulations and have been succesful in reducing cigarette smoking, hookah smoking has not decreased significantly due to inappropriate and inadequate consideration1. The first global health treaty was approved by 179 countries worldwide, the results of which indicated that laws controlling tobacco consumption do not adequately address hookah smoking^39,53^. Participants in another study stated that government legislation could be a significant step toward reducing the prevalence of hookah smoking^54^. In a review of the laws in 62 countries, only the United States, United Kingdom, India, United Arab Emirates and Pakistan had passed influential laws controlling hookah smoking^55^.

Selling or serving tobacco products including hookahs in traditional restaurants, and hookah smoking in establishments with traditional music, can attract people to these places and add to their interest in smoking hookahs^56^. Therefore, programs for the prevention of hookah smoking in society would require certain planning and policy making to eliminate such places. It is also recommended that higher taxation on tobacco and raising its price should be considered.

### Strengths and limitations

A strength of the present research was the extraction of determining factors of hookah smoking within a certain framework with five separate levels (intrapersonal, interpersonal, organizational, social, political). Moreover, interventions are suggested for each level of determinants in light of definite relevant theories. The present research is the first study to look into the determinants of hookah smoking among women. Moreover, the present findings can form the basis for future qualitative and quantitative studies and aid in the design of more effective interventions to reduce the rate of hookah smoking among women and girls.

There were also some limitations in the present systematic review. First, only articles in English or Persian were included. Another limitation was the absence of experimental studies and longitudinal data on hookah tobacco smoking. Also, the causality and temporal relationships between the variables cannot be determined in such studies.

## CONCLUSIONS

According to the present findings, hookah smoking among women is multifactorial. Thus, the preventive interventions should be multilevel and address different risk factors in various domains, especially at personal and environmental levels. A positive attitude at the intrapersonal level, subjective norms at the interpersonal level, low cost and easy access to hookah at the organizational level, cultural norms and acceptance at societal level, and absence of law at the political level correlated with hookah consumption among women. Changing the positive attitude toward hookah by women, learning to resist friends’ pressure to smoke, highlighting the unpleasantness of hookah smoking by segregating places with transparent walls within public places, showing hookah smoking as socially unacceptable in the virtual world, limiting access to hookah tobacco products, and effectively implementing rules that restrict hookah smoking in public places, can help reduce hookah consumption. This review suggests that more qualitative and quantitative research needs to be conducted to look into environmental factors (political and economic). For more effective interventions, the determinants of quitting smoking must be ascertained through interviews with women who have permanently or temporarily stopped smoking. The present review further suggests that more interventional, observational and longitudinal investigations be conducted to precisely identify the determinants of hookah smoking.
