# Supplementary material for: Factors associated with hookah smoking among women: A systematic review
Source: Tob Prev Cessat. 2019 Aug 1;5:26. doi: 10.18332/tpc/110586 (PMC7205165; doi:10.18332/tpc/110586)
Supplement: Supplementary file 1 [file TPC-5-26-s1.pdf]

## Results of the quality assessment for the qualitative studies ($n = 7$)

| Study | Date/Author | Abstract/title | Introduction/aims | Data collection | Sampling | Analysis | Ethics/bias | Results | Generability | Implications | Total | Grade |
|---|---|---|---|---|---|---|---|---|---|---|---|---|
| Factors that Contribute in the First Hookah Smoking Trial by Women: A Qualitative Study from Iran | Baheiraei et al /2015 | 4 | 3 | 3 | 3 | 3 | 4 | 3 | 3 | 2 | 28 | B |
| Qualitative Studies Smoking Hookah Among Girls And Young Women | Sohrab Zade et/ 2015 | 3 | 4 | 3 | 4 | 3 | 1 | 4 | 2 | 3 | 27 | B |
| Thematic analysis focus group discussions (FGDs) and key informant interviews | Rima Afifi et/2013 | 4 | 3 | 4 | 4 | 4 | 4 | 4 | 4 | 3 | 34 | A |
| find the role of psycho-social needs and gaps as a possible risk factor for hookah smoking initiation in women | Baheiraei et al/2015 | 3 | 4 | 4 | 4 | 4 | 4 | 3 | 4 | 4 | 34 | A |
| The rise in narghile (shisha, hookah) water pipe tobacco smoking: A qualitative study of perceptions of smokers and non-smokers | Rima T Nakkash et/2011 | 4 | 3 | 4 | 4 | 4 | 4 | 4 | 4 | 4 | 35 | A |
| Water pipe (Hookah) Smoking Among Youth And Women In Canada Is New Not Traditional | Fadi Hammal et al/2015 | 3 | 3 | 3 | 4 | 4 | 3 | 4 | 4 | 1 | 29 | B |
| role of family members' smoking behaviours as a possible risk factor for initiation of hookah smoking in women | Baheiraei/2015 | 3 | 4 | 4 | 4 | 3 | 4 | 4 | 3 | 1 | 30 | A |

© 2019 Dadipoor S.
