# Supplementary material for: Factors associated with hookah smoking among women: A systematic review
Source: Tob Prev Cessat. 2019 Aug 1;5:26. doi: 10.18332/tpc/110586 (PMC7205165; doi:10.18332/tpc/110586)
Supplement: Supplementary file 2 [file TPC-5-26-s2.pdf]

STROBE Statement—Checklist of items that should be included in reports of *cross-sectional studies* (n=8)

Authors	Title	Date/ Country	Title and abstract	Background/rationale	Objectives	Study design	Setting	Participants	Variables	Data sources/ measurement	Bias	Study size	Quantitative variables	Statistical methods	Participants	Descriptive data	Outcome data	Main results	Other analyses	Key results	Limitations	Interpretation	Generalisability	Funding	Grade
Nargis Labib	Comparison of cigarette and water pipe smoking among female university students in Egypt	2007 Egypt	*	*	*	-	*	-	-	*	-	-	*	*	-	*	*	-	*	*	-	*	-	-	12
Kalid Yunis et al	Patterns and predictors of tobacco smoking cessation: A hospital-based study of pregnant women in Lebanon	Beirut, Lebanon survey 2007	*	*	*	*	*	*	-	*	-	-	*	*	*	*	*	-	*	-	*	*	*	*	17
N. S. Dar-Odeh et al	Narghile Smoking among Jordanian Educated Working Women: Attitudes and Beliefs	Amman, Jordan 2013	*	*	*	*	*	-	-	*	-	-	-	*	*	*	*	-	*	-	-	-	-	-	11
Firoozabadi et al	Predicing Factors on Coninued Intenion of	Iran 2015	*	*	*	*	*	*	-	*	-	*	-	*	-	*	*	-	*	*	*	-	*	*	16

	Waterpipe Smoking Among Women in Bushehr																							
M. Chaaya et al	Knowledge, attitudes, and practices of argileh (water pipe or hubble-bubble) and cigarette smoking among pregnant women in Lebanon	Lebanon 2004	*	*	*	*	*	*	-	*	-	*	-	*	-	*	*	-	*	*	*	*	*	-	16
W.M ziak,et al	Gender and smoking status-based analysis of views regarding waterpipe and cigarette smoking	Syria 2004	*	*	*	*	*	*	-	*	-	-	-	*	-	*	*	-	*	*	*	*	*	*	16
Mohammed Azab et al	Exposure of Pregnant Women to Waterpipe and Cigarette Smoke	Jordan 2013	*	*	*	*	*	-	*	*	-	-	*	*	*	*	*	-	*	*	*	*	-	*	17
Pascale Salameh et al	Lower Prevalence of Cigarette and Water pipe Smoking, But a Higher Risk of Waterpipe Dependence in Lebanese Adult Women Than in Men	Lebanon 2012	*	-	*	*	*	*	*	*	-	*	*	*	*	*	*	-	*	*	*	*	*	-	18

© 2019 Dadipoor S.
